# Critical care management of right ventricular failure in pediatric left ventricular assist devices: An advanced cardiac therapies improving outcomes network (ACTION) endorsed statement

**DOI:** 10.1016/j.jhlto.2025.100361

**Published:** 2025-08-05

**Authors:** Edon J. Rabinowitz, Nancy Ghanayem, Lydia Wright, Amee M. Bigelow, Shilpa V. Govardhan, Tara M. Neumayr, Tanya Perry, Meghna D. Patel, Robert A. Niebler, Kevin P. Engelhardt, Isaura Diaz, Joseph Philip, Arene Butto, Iki Adachi, David M. Peng, David Kwiatkowski, Sebastian C. Tume

**Affiliations:** a Washington University School of Medicine in St Louis, Division of Pediatric Critical Care Medicine, Department of Pediatrics and St Louis Children’s Hospital, St. Louis, MO; b University of Chicago, Section of Pediatric Critical Care, Department of Pediatrics, Chicago, IL; c Department of Pediatrics, The Heart Center, Nationwide Children's Hospital, The Ohio State University, Columbus, OH; d Rady Children’s Hospital, University of California San Diego, San Diego, CA; e Division of Pediatric Critical Care, Cohen Children’s Medical Center of New York, Northwell Health; f Stanford University School of Medicine, Division of Pediatric Cardiology, Palo Alto, CA; g Medical College of Wisconsin, Department of Pediatrics and Children's Wisconsin, Herma Heart Institute, Milwaukee, WI; h Phoenix Children’s, Division of Pediatric Critical Care, Phoenix, AZ; i Vanderbilt University Medical Center, Department of Pediatrics, Division of Pediatric Critical Care, Nashville, TN; j University of Florida Health, Department of Pediatrics, Division of Pediatric Cardiology, Jacksonville, FL; k Children’s Healthcare of Atlanta, Division of Pediatric Cardiology, Atlanta, GA; l Division of Critical Care, Department of Pediatrics, Baylor College of Medicine and Texas Children’s Hospital, Houston, TX; m Division of Pediatric Cardiology, Department of Pediatrics, University of Michigan Medical School, Ann Arbor, MI

**Keywords:** Pediatric cardiac critical care, Right ventricular failure, Subpulmonary failure, Mechanical circulatory support, Left ventricular assist device, Guide, Mechanical ventilation

## Abstract

The care of the right ventricle (RV) following left ventricular assist device (LVAD) implantation remains a major clinical challenge, with right ventricular failure (RVF) contributing significantly to morbidity and mortality. While much of the literature focuses on preoperative risk stratification and long-term management, there is limited guidance on the immediate postoperative period from a critical care perspective, particularly in pediatric patients. This review aims to provide practical guidance on the bedside management of the RV in the perioperative period following LVAD implantation in children with biventricular circulation, offering a framework for optimizing RV function and preventing failure. We discuss the pathophysiology of RVF in this setting, highlight key hemodynamic principles, and explore targeted interventions including volume management, inotropic and pulmonary vasodilator support, ventilatory strategies, mechanical circulatory support options, and strategies to mitigate secondary organ dysfunction. By addressing these pediatric-specific critical care considerations, we aim to assist bedside providers in optimizing outcomes for children undergoing LVAD implantation.

## Introduction

More than 50% of children undergoing isolated left ventricular assist device (LVAD) implantation may develop right ventricular failure (RVF), an important cause of morbidity and mortality.^1^ Post-LVAD RVF is associated with survival rates below 50%.^2^, ^3^, ^4^ A deeper understanding of RVF in the context of LVAD support is essential for effective management. This review provides critical care expert consensus on diagnosis, risk stratification, clinical management, and prevention strategies to optimize RVF outcomes in the postoperative period of children with biventricular circulation.

The right ventricle (RV) is uniquely adapted to tolerate acute preload changes due to its anatomical configuration, which allows significant volume expansion without excessive wall stretch. However, RV performance is limited by its sensitivity to increased afterload and by the competence of the tricuspid valve.^5^ Ventricular interdependence, arising from the shared septal wall, common myocardial fibers, and single pericardial enclosure, highlights the dynamic interplay between the ventricles. Systolic and diastolic ventricular interactions under normal and pathological conditions have been extensively reviewed.^6^, ^7^, ^8^, ^9^, ^10^, ^11^, ^12^

In systolic left ventricular (LV) failure, elevated LV filling pressures lead to increased RV pressures.^13^ LVAD implantation reduces LV end-diastolic volume and pressure, lowers pulmonary capillary wedge pressure, and repositions the interventricular septum (IVS) to midline.^14^ These changes decrease RV end-diastolic pressures, increase RV end-diastolic volume, and reduce the IVS contribution to RV systole.^15^ This altered interventricular dependence is vulnerable to acute increases in RV preload or afterload, contributing to RV dysfunction after LVAD implantation.

### Definitions and assessment of right ventricular failure

RVF is defined by the RV’s inability to fill or eject blood effectively despite adequate preload, impairing pulmonary circulation and left heart filling.^16^, ^17^, ^18^ Post-LVAD RVF is typically identified using clinical and hemodynamic criteria, including the need for prolonged inotropic support, RV afterload reduction, or RVAD use.^19^ Clinically, RVF manifests with reduced cardiac output and systemic venous congestion, leading to progressive organ dysfunction, particularly hepatic, renal, and splanchnic failure, if unaddressed.^20^, ^21^ Hemodynamic indicators include elevated CVP (>14 mmHg), increased oxygen extraction (decreased mixed venous oxygen saturation), and low cardiac index (<2.2 L/min/m²), all of which compromise LVAD preload.^22^, ^23^, ^24^

In adult populations, increasing efforts have aimed to distinguish RVF from LVF to highlight their distinct pathophysiologic implications.^29^ The 'right heart circulatory system' extends from the systemic veins to the pulmonary capillary bed, with RVF defined by elevated systemic venous pressures and/or insufficient forward flow into the pulmonary circulation.^29^ Key hemodynamic indices in adults include the right ventricular stroke work index (RVSWI), which assesses RVF via right heart catheterization,^25^, ^26^, ^30^, ^31^, ^32^ and the pulmonary artery pulsatility index (PAPi), defined as the ratio of pulmonary artery pulse pressure to right atrial pressure, a superior predictor of RVF in LVAD patients.^27^, ^28^, ^32^, ^33^, ^34^, ^35^ However, these definitions are inconsistently applicable in pediatrics due to limitations of the existing literature, which is largely based on low-powered, single-center, retrospective studies that rarely focus specifically on RVF in the context of pediatric LVAD. This contributes to variability in clinical practice, although some advocate that pre-implant PAPi and RVSWI may serve as a meaningful predictor of post-LVAD RVF in select pediatric patients.^36^, ^37^

RVF post-LVAD can occur at different stages of recovery, driven by underlying cardiomyopathy, altered loading conditions, and septal shifts (Table 1).^38^, ^39^ Hyperacute RVF presents immediately as failure to wean from cardiopulmonary bypass,^18^ acute RVF develops within 30 days with dependence on inotropic or pulmonary support,^21^ and chronic RVF emerges beyond 30 days, characterized by low LVAD flow, elevated CVP, and organ dysfunction potentially exacerbated by valvar issues or conduction disturbances. The Advanced Cardiac Therapies Improving Outcomes Network (ACTION) adverse event definition includes hemodynamic and imaging criteria indicating elevated RVEDp and RV dysfunction ≥10 days post-LVAD implantation (Table 2).^24^

**Table 1:** Categorization of Right Ventricular Failure (RVF) After Left Ventricular Device (LVAD) Implantation by Timing of Onset

Hyperacute Intra-operative	Acute <30 days post-implant	Chronic >30 days post-implant
o Inability to separate from CPB due to inadequate right ventricular function. o Inadequate filling of the LVAD. o Inability to achieve adequate LVAD flow despite optimization attempts o Dependence on pharmaceutical support and resuscitation. o Presence of hemodynamic, laboratory, and echocardiographic evidence of RVF. o May require additional mechanical circulatory support for the RV.	o Defined by clinical, laboratory, echocardiographic, and hemodynamic disturbances. o Inability to wean from vasoactive and inotropic support or inhaled nitric oxide (iNO). o Continued suboptimal filling of the LVAD.	o Acute or ongoing suboptimal LVAD flow and fill. o Ongoing elevation of CVP (>10−14 mmHg) or ascites requiring aggressive fluid balance management. o Requirement of vasoactive or inotropic support. o Suboptimal mixed venous oxygenation. o Ongoing end organ injury.

CPB; cardiopulmonary bypass, LVAD; Left Ventricular Assist Device, RVF; Right Ventricular Failure, iNO; inhaled nitric oxide, CVP; central venous pressure.

**Table 2:** Advanced Cardiac Therapies Improving Outcomes Network (ACTION) consensus definition of Right Ventricular Failure (RVF) after Left Ventricular Device (LVAD) Placement^1^.

	Description
Definition	Only in biventricular circulation **Both** hemodynamic and imaging categories Present ≥10 days post-index operation, without device malfunction or thrombus. Alternatively, criteria are met at any time if RVAD or ECMO is required.
Hemodynamics	Right-sided or systemic venous filling pressure >14 mm Hg for >24 h OR clinical signs of high CVP (effusions, ascites, hepatomegaly) with inadequate LVAD preload (CO <50% expected, unresponsive to pump changes)
Imaging	Moderate/severe tricuspid regurgitation OR Moderate/severe RV systolic dysfunction
Grading	**Grade 1 & 2:** Not applicable. **Grade 3:** Requires vasoactive infusions, new invasive effusion/ascites management, or prolonged surgical drain use due to right heart dysfunction ≥10 days post-index procedure. **Grade 4:** ECMO for right heart dysfunction or RVAD implantation as a separate procedure. **Grade 5:** Death directly or primarily due to right heart dysfunction.

RVAD; Right Ventricular Assist Device, LVAD; Left Ventricular Assist Device, TR; Tricuspid Regurgitation, RV; Right Ventricle, CVP; Central Venous Pressure, ECMO; Extracorporeal Membrane Oxygenation

#### Echocardiographic assessment

Echocardiography is vital for evaluating RVF pathophysiology. Key indicators include RV dilation, septal shift, tricuspid regurgitation (TR), increased RV/LV ratio, reduced tricuspid annular plane systolic excursion (TAPSE), IVC dilation without respiratory variation, and impaired RV fractional area change.^19^, ^21^, ^24^, ^40^, ^41^ Preexisting RV systolic dysfunction does not always predict post-LVAD RVF, highlighting the need to assess findings such as tricuspid incompetence and acute septal shifts.

#### Hemodynamic monitoring

Appropriate hemodynamic monitoring, including CVP and right/left atrial pressures, should be considered before and after LVAD implantation. Pulmonary artery catheters may be useful in adolescents to assess RV and LV loading conditions.^5^ Postoperative arterial pressure pulsatility reflects native LV ejection contributions to total cardiac output alongside the LVAD, while LVAD output estimates cardiac index in non-pulsatile flow patients. Non-invasive techniques like near-infrared spectroscopy (NIRS) provide additional insights into oxygen delivery and consumption balance.

#### Laboratory monitoring

Laboratory tests, such as lactate, venous oximetry, liver enzymes (AST, ALT), bilirubin, lipase, creatinine, and NGAL, offer critical information on oxygen utilization and organ recovery.^41^, ^42^, ^43^, ^44^ Biomarkers like BNP and NT-proBNP are particularly sensitive for detecting RV dysfunction.^41^, ^43^, ^45^

### Optimization of right ventricular function before LVAD placement

Predictors of RVF post-LVAD implantation offer moderate predictive value for clinical management (Table 3).^42^ Clinical risk factors include pre-implant mechanical ventilation, renal replacement therapy, and female gender.^42^ Pediatric-specific predictors include younger age, smaller body size, and chemical paralysis during the first week post-LVAD. An INTERMACS score of 1 is linked to early RVF, while pulsatile flow devices are associated with late RVF.^1^ Smaller studies on Berlin Heart devices (Berlin Heart, Berlin, Germany) highlight prior ECMO use and end-organ dysfunction as additional risks.^43^ Echocardiographic markers of severe RV dysfunction in adults may predict post-LVAD RVF,^46^, ^47^, ^48^, ^49^ but no single biomarker reliably predicts its occurrence. Comprehensive assessments integrating clinical, hemodynamic, and biochemical data are essential to identify high-risk patients.

**Table 3:** Predictors of Right Ventricular Failure (RVF) Before Left Ventricular Device (LVAD) Placement

Predictors of Right Heart Failure	Description
1. Technology Support Requirements prior to LVAD implantation	Need for mechanical ventilatory support, renal replacement therapies.
2. Patient Demographics	Higher risk in females, younger age, smaller size.
3. Clinical Indicators	Chemical paralysis during the first week post-LVAD; severity of illness score (INTERMACS score of 1) prior to implant.
4. (Adult) Echocardiographic Parameters	Severe TR, RV fractional area change indicating RV function <30%, RV strain with peak cut off −9.6%, TAPSE <7.5 mm, increased RV to LV end diastolic diameter.
5. Other Risk Factors	Preimplant ECMO, end organ dysfunction.

LVAD; Left Ventricular Assist Device, INTERMACS; Interagency Registry for Mechanically Assisted Circulatory Support, TR; Tricuspid Regurgitation, RV; Right Ventricle, TAPSE; Tricuspid Annular Plane Systolic Excursion, LV; Left Ventricle, ECMO; Extracorporeal Membrane Oxygenation

Preoperative management of RV systolic dysfunction focuses on optimizing preload, afterload, and contractility. Preload should be carefully managed to avoid volume overload, which can lead to systemic venous hypertension and pulmonary congestion. Diuretics should be used judiciously with close monitoring of CVP and markers of cardiac output. Myocardial contractility can be supported with inotropes like PDE3 inhibitors or beta-adrenergic agonists, while vasoactive agents are used cautiously due to their potential to increase systemic afterload and elevate LVEDP.

RV afterload reduction often includes inhaled nitric oxide (iNO) in the acute phase to lower pulmonary vascular resistance (PVR) but should be used cautiously in the presence of elevated LV filling pressures, as its clinical impact remains uncertain.^50^ Long-acting pulmonary vasodilators should be used cautiously, as they may exacerbate pulmonary edema in the setting of elevated LVEDP. Respiratory optimization is critical and discussed separately.

The primary goal of pre-operative critical care management is to optimize existing circulation and preserve end-organ function in preparation for LVAD implantation. Intraoperatively, myocardial protection, minimizing cardiopulmonary bypass aortic cross clamp times, precise LVAD titration, and minimizing blood transfusions are vital for achieving optimal outcomes.^44^, ^51^

### Management of RVF after LVAD implant

**Figure 1:**
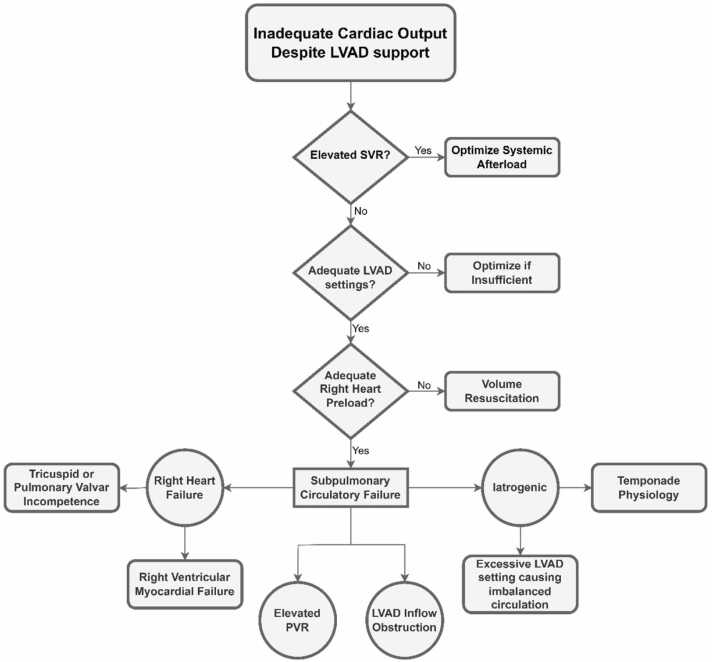
Pathway Considerations for Assessment and Management of Inadequate Cardiac Output in the Presence of LVAD Support. Begin by assessing systemic vascular resistance (SVR) to determine the need for afterload-reducing agents. If LVAD afterload is optimized, evaluate the LVAD settings to ensure adequate support. If the settings are insufficient, consider adjustments to improve LVAD output (note that in the presence of significant aortic insufficiency, higher settings may exacerbate a circular LVAD shunt). If settings are adequate, assess right heart preload status and determine if volume resuscitation is required. If volume is sufficient, consider the possibility of subpulmonary circulatory failure, which may be caused by right heart failure (e.g., RV myocardial failure, tricuspid or pulmonary valve incompetence), elevated pulmonary vascular resistance (PVR), mechanical obstruction to LVAD inflow, tamponade physiology, or excessive LVAD settings leading to an imbalanced circulation (e.g., collapsed left heart as compared to dilated right heart due to excessive septal shift).

LVAD placement alone may improve RV function by reducing LA pressure and RV afterload through adequate LV decompression. However, RV failure might still occur in up to 20–40% of cases post-LVAD,^52^, ^53^, ^54^, ^55^ often necessitating additional support strategies. Pediatric cardiac intensivists are well equipped in management of RV dysfunction frequently seen in setting of congenital heart disease. Management of RVF in setting of LVAD is primary focused on recognizing and addressing subpulmonary circulatory failure of various causes (Figure 1). With that in mind the management approach to RVF not only involves optimization of loading conditions and contractility but also a careful adjustment of LVAD support relative to existing RV performance. Importantly, one must pay close attention to and avoiding iatrogenic imbalanced circulation which could result in compromised LVAD preload and LVAD performance further exacerbating RVF (i.e. excessive decompression of the left heart coupled with right heart dilation due to increased systemic venous return).

#### RV preload considerations

In early post operative phase volume resuscitation might be necessary to achieve optimal preload in setting of fluid shifts. Finding an optimal point of RV preload is crucial to avoid RV over-distension which can lead to geometrical changes resulting in compromised systolic function, exacerbate tricuspid regurgitation, further worsening RV volume overload. Timely initiation of diuresis is essential to avoid the above mentioned pathophysiology and this should be attempted after resolving post operative bleeding, metabolic acidosis and postoperative capillary leak.^51^ An initial target CVP of 8–12 mmHg, urine output >1 mL/kg/hour, and maintenance of adequate renal perfusion pressure provide a reasonable starting framework. Vasoactive agents may be used to support renal perfusion as needed, but care must be taken to avoid excessive systemic afterload that may impair LVAD performance. Other clinical tools such as physical exams, respiratory variation, device filling, and weight trends are equally important for accurate fluid balance assessment. Sedation, paralysis, and positive pressure ventilation (PPV) also influence preload.^56^

Patients with hypertrophic or restrictive cardiomyopathy undergoing LVAD placement may have increased sensitivity to changes in these parameters, requiring tailored strategies that are beyond the scope of this review.

#### RV afterload considerations

A major component of RV afterload is PVR. Avoiding hypoxia, acidosis, and hypercapnia is crucial and use of PPV should aim to maintain FRC avoiding alveolar under- or overdistension. iNO is the first line agent recommended for those with documented pulmonary hypertension based on perioperative assessment^57^, ^58^, ^59^ and can also be used prophylacticaly in those at high risk of elevations in pulmonary pressures. Agents such as inhaled iloprost or epoprostenol may be considered for chronic or refractory hypertension recognizing their side effects of hypotension and platelet dysfunction. Phosphodiesterase-5 (PDE-5) inhibitors can facilitate transitioning from prolonged iNO therapy.^60^ As many of these therapies are initiated intraoperatively, close collaboration among critical care, transplant, pharmacy, and cardiothoracic surgery is essential to delineate their effects and long term needs.

Milrinone and isoproterenol can also reduce RV afterload and thus enhance RV stroke volume, improving LVAD output.^61^, ^62^ Use of these agents requires monitoring of filling pressures, oxygen extraction markers, and clinical signs like urine output, feeding intolerance, and end organ function.

#### Tricuspid regurgitation considerations

TR affects RV performance by impairing antegrade blood flow. Preoperative assessment must include TR severity and the consideration for need of valvular intervention. While LVAD patients often undergo cardiopulmonary bypass, the additional time for tricuspid repair or replacement must be weighed against bypass and cross-clamp risks. TR caused by annular dilation may improve with reduced LA pressure and effective fluid management, though structurally abnormal valves often remain regurgitant post-LVAD. Managing significant TR post-LVAD involves careful volume management and afterload optimization. However persistent hemodynamically significant TR marked by systemic venous congestion, CVP >14 mmHg, ongoing RV dilation, and diuretic dependence, may necessitate reoperation for tricuspid valve intervention.

### Acute kidney injury and cardiorenal syndrome in pediatric LVAD patients

Acute kidney injury (AKI) is common in pediatric cardiac surgery and predicts morbidity, mortality, and progression to chronic kidney disease (CKD) in severe cases.^63^, ^64^, ^65^ Contributing factors include RVF, low cardiac output, endothelial dysfunction post-CPB, and nephrotoxin exposure.^63^, ^66^ Cardiorenal Syndrome (CRS), a reciprocal relationship between heart and kidney dysfunction, is particularly relevant in right heart failure. Venous congestion post-LVAD, with increased systemic blood flow and renal venous hypertension, exacerbates CRS and AKI.^41^, ^67^ Inflammatory activation during RVF and LVAD support further worsens CRS.^67^, ^68^ Although renal function often improves post-LVAD, long-term outcomes with continuous-flow devices remain unclear.^69^, ^70^ Failure to normalize renal function on LVAD, or pre-transplant renal disease, heightens CKD risk post-transplantation.^71^

Post-LVAD fluid management is crucial for balancing organ perfusion and preventing venous congestion. While pediatric-specific evidence is limited, guidelines suggest isotonic fluids with dextrose and potassium.^64^, ^72^, ^73^, ^74^ Goal-directed therapy should optimize RV preload while avoiding overload, guided by arterial pressure, CVP, urine output, weights, biochemical markers, and regional oximetry. Optimal CVP (8–10 mmHg) in children requires individualized assessment.^68^, ^75^, ^76^ Prolonged systemic venous hypertension reduces renal and splanchnic perfusion leading to renal dysfunction.^71^ As pediatric renal perfusion thresholds are poorly defined, careful hemodynamic evaluation is essential.

CRS diagnosis combines renal biomarkers, imaging, and functional testing. Serum creatinine, while commonly used, is a late marker and may be unreliable in chronic heart failure due to muscle wasting. Early biomarkers like cystatin C, albuminuria, and BNP are stronger mortality predictors.^63^, ^66^ Renal Doppler ultrasonography links intrarenal venous flow with elevated right atrial and central venous pressures.^66^, ^67^, ^71^, ^77^ Elevated intra-abdominal pressure is another negative prognostic factor, though pediatric thresholds are undefined.^65^, ^78^ The Furosemide Stress Test predicts renal function post-operatively but its pediatric post-implant applicability remains unclear.^79^, ^80^, ^81^

Treatment of CRS prioritizes decongestion and renal support (Table 4). Loop diuretics are first-line, with continuous infusion more effective than boluses.^82^, ^83^, ^84^ High-dose diuretics may alleviate symptoms without significant renin-angiotensin-aldosterone activation.^66^ Vasodilators like nitroprusside may address diuretic resistance by mitigating renal afferent or arteriolar vasoconstriction seen in chronic heart failure.^85^, ^86^, ^87^ Extracorporeal renal replacement therapies (CRRT) are effective for oliguric renal failure and severe fluid overload, though patient selection and timing remain unclear.^88^, ^89^ Preemptive CRRT catheter placement should be considered during surgery to enable early initiation in high-risk patients. For paracorporeal LVADs (e.g., EXCOR Berlin Heart), connecting cannulas to an ECMO circuit with ultrafiltration allows aggressive fluid management. Peritoneal dialysis is another option, offering ease of catheter placement and intra-abdominal pressure reduction, though potential driveline interference must be considered.^78^, ^90^

**Table 4:** Cardiorenal Syndrome (CRS) Treatment Strategies

CRS Treatment Strategy	Details
Loop Diuretics	Remain the primary treatment for CRS. Continuous infusion is more effective than bolus doses for decongestion. High-dose (2.5x home dose) does not increase RAAS activation and may improve symptoms.
Diuretic Resistance	Mitigated by vasodilators like nitroprusside. Early high-dose continuous infusion diuretics, monitoring for resistance, and escalating therapy is recommended.
Extracorporeal Ultrafiltration (UF)	Used for diuretic-refractory patients. The timing for initiation is unclear, and early studies in adults showed mixed results, warranting further pediatric-specific research.
Continuous Kidney Support Therapy (CKST)	Requires multidisciplinary care with vascular access challenges in pediatric LVAD patients. Catheter placement during surgery may benefit high-risk patients.
Peritoneal Dialysis (PD)	Viable for LVAD patients. PD offers ease of catheter placement and alleviates intra-abdominal pressure from fluid collections.

CRS; Cardiorenal Syndrome, RAAS; Renin-Angiotensin-Aldosterone System, UF; ultrafiltration, CKST, Continuous Kidney Support Therapy, LVAD; Left Ventricular Assist Device, PD; Peritoneal Dialysis.

### Pharmacological management

**Figure 2:**
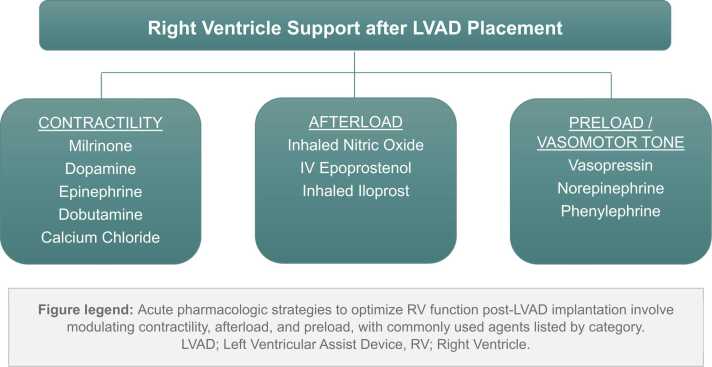
Peri-Operative Pharmacological Support Strategies for the Right Ventricle (RV) After Left Ventricular Assist Device (LVAD) placement.

Post-LVAD pharmacological support is crucial for maintaining RV function by optimizing preload, inotropy, and afterload (Figure 2). Common inotropes include milrinone, epinephrine, and dopamine. No single agent has proven superior,^1^, ^43^, ^91^, ^92^, ^93^, ^94^, ^95^ and excessive doses should be avoided to prevent tachycardia and increased systemic vascular resistance (SVR) that can impair LVAD function.

Milrinone, the most commonly used inodilator, offers both inotropic and vasodilatory effects but requires close monitoring for side effects, particularly in patients with renal or liver dysfunction or those prone to vasomotor instability.^96^ Importance of coronary perfusion should be also recognized with focus on optimized mean arterial and central venous pressures. During CRS or renal replacement therapy, hypotension may necessitate vasopressors like vasopressin, angiotensin agonist, or norepinephrine, guided by center protocols.^97^, ^98^, ^99^ These agents should be reserved for vasoplegia-induced hypotension to avoid increased SVR and compromised LVAD flow.

Post-LVAD arrhythmias, common in the early postoperative period, include SVT and VT with occurrences of 15% each in single-center studies.^100^ Ventricular arrhythmias are linked to higher mortality in pediatric patients on LVAD.^100^ Untreated arrhythmias impair RV function, reduce LVAD preload, and compromise output. Management includes electrical or intravenous cardioversion and antiarrhythmic therapy with agents such as amiodarone, sotalol, or procainamide. Refractory arrhythmias should be co-managed with electrophysiologists, as pacemaker support may be required.

Given the dynamic nature of RV support, careful monitoring and individualized medication adjustments are essential during early postoperative recovery, particularly in response to fluid shifts, changes in positive pressure ventilation, and increased metabolic demands associated with sedation weaning and mobilization. In the absence of standardized dosing protocols, therapies should be tailored to optimize hemodynamics while minimizing side effects (Table 5).

**Table 5:** Indications for Medication Titration for Right Ventricle (RV) Support After Left Ventricular Assist Device (LVAD) Placement

Indications to Increase RV support	Indications to Wean RV support
o Poor LVAD fill o Hepatomegaly o Elevated central venous pressure o Poor urine output o Tachycardia o Poor RV function on echocardiography o Worsening liver function or transaminases o Ascites / Chest tube output / Pleural effusions	o Low central venous pressure o Well-functioning LVAD o Improving RV function on echocardiography o Improving renal health

LVAD; Left Ventricular Assist Device, RV; Right Ventricle.

### Mechanical ventilator management

Optimization of the respiratory system is crucial in successful management of post-LVAD RVF. Use of PPV requires clear understanding of cardiopulmonary interactions and their effect on RV preload and afterload. PPV can also influence LVAD function and IVS position, especially during transitions on and off PPV. Ventilator management for RVF depends on its underlying pathophysiology, including septal involvement, systolic RV dysfunction, or volume overload due to valvular regurgitation. As example positive intrathoracic pressure under continuous-flow LVAD support may improve RV efficiency by offsetting RV stroke work, but this benefit can reverse post-extubation due to ventilation-perfusion imbalances.^101^ Thus, effective ventilator strategies must balance optimizing RV preload, PVR, and LV afterload to improve RV stroke volume.

Ventilation may further affect PVR through changes in pH, oxygen levels, and lung volumes. In the presence of a reactive pulmonary vascular bed, alkalosis and hyperoxia reduce PVR, while acidosis and hypoxia increase PVR. Elevated PVR increases RV impedance, reducing RV ejection impairing LV filling and LVAD performance all of which underscores the importance of precise PPV titration. Optimal PVR is achieved near FRC, requiring careful lung volume adjustments to avoid underinflation, hypoxia, or overdistension.^102^

#### Ventilatory strategies

Post-LVAD ventilator management should align with patient pathophysiology. Chronic ventilatory patients require gradual weaning, while those intubated for LVAD implantation often progress faster (Table 6). LVAD support can facilitate quicker weaning, especially if mechanical ventilation was used to unload the LV and respiratory muscles. Prolonged ventilation is more common in patients with pre-LVAD ventilator use, ECMO exposure, or significant valvular regurgitation.^103^ Improved circulation alone may not resolve chronic airway or lung disease, necessitating maintenance of pre-LVAD settings. High PEEP, causing lung over-distention, should be avoided as it can lower systemic venous return and impair LVAD performance.^104^ In patients who fail weaning, residual conditions such as left atrial hypertension, diaphragm paralysis, or lung injury should be investigated. Rehabilitation is essential for addressing weakness-related delays (Table 7).

**Table 6:** Mechanical Ventilation Approach For Those On Chronic Respiratory Support

Category	Patients with MV Support Prior to LVAD Implantation
Preoperative MV Considerations	Requires careful preoperative evaluation for post-op ventilator management.
Reasons for MV Support	Acute lung injury (pulmonary edema, infection, ARDS), chronic lung disease, upper airway anomalies, circulatory/metabolic support, sedation.
Impact of Previous ECMO	Patients with prior ECMO encounter are at double the risk of prolonged MV post-LVAD.
Risk Factors for Prolonged MV	Moderate/severe tricuspid regurgitation, moderate/severe mitral regurgitation, prior ECMO support.
Chronic Lung Disease/Structural Airway Disease	Chronic lung disease or airway anomalies require continued management as pre-LVAD.
PEEP Considerations	High PEEP (e.g., in tracheomalacia) can negatively affect LVAD performance (decreased cardiac output).
Weaning from MV	Weaning can be more challenging in patients with chronic disease; careful monitoring required.
Single-Ventricle Considerations	Single-ventricle patients (Glenn/Fontan) can be negatively impacted by positive pressure ventilation.
Postoperative Ventilator Management Focus	Continued support for chronic lung disease, tailored to maintain LVAD functionality.
Primary Goals of Ventilation Support	To reduce metabolic demands, provide respiratory and circulatory support.

LVAD; Left Ventricular Assist Device, MV; Mechanical Ventilation, ARDS; Acute Respiratory Distress Syndrome, CPB; Cardiopulmonary Bypass, ECMO; Extracorporeal Membrane Oxygenation, PEEP; Positive End Expiratory Pressure, VILI; Ventilator Induced Lung Injury.

**Table 7:** Evaluating Extubation Readiness After Left Ventricular Assist Device (LVAD) Placement

Pre-Extubation Clinical Status	Criteria/Considerations
Adequacy of Gas Exchange	• Maintain SpO₂ >92% (adjust for single-ventricle patients). • Target FiO₂ <50% and PEEP <7. • Assessment of OI, PaO2/FiO2 ratio and A-a gradient should be used to diagnose oxygenation impairments. • CO2 retention during weaning trials might suggest muscle pump weakness or diaphragmatic impairment • If pulmonary edema persists, assess LV unloading. • Evaluate for inflow/outflow obstruction if lung congestion persists. • If imaging is inconclusive, consider catheterization for direct pressure measurements to refine pump settings.
Hemodynamic Stability	• Optimal CO is required to support spontaneous breathing. Optimize LVAD support to achieve optimal cardiac output and perfusion • Use careful approach to weaning of supportive therapies before or after extubation especially in a setting of marginal circulation.
Sedation Status	• Objective scores should be used to optimize sedation prior to extubation to prevent oversedation. • Weaning regimens should be designed in collaboration with clinical pharmacist to minimize withdrawals which can lead to increased oxygen demands. • Careful monitoring of delirium should be done through use of objective scores
Neurologic Events and Residual Lesions	• Conduct neurologic assessment and ensure adequate respiratory drive, especially after recent injury. • In high risk patients it might be reasonable to attempt extubation but anticipate possible need for chronic airway support. • Identify causes of extubation failure to accurately guide long-term support decisions. • Consider role of antiepileptic medications and their sedative effects especially during up titrations.
Nutritional State	• Malnutrition and debilitation are common in heart failure patients thus nutritional status should be carefully assessed and optimized before extubation especially in young infants. • Ensure adequate energy intake to support spontaneous breathing and maintain muscle strength. • Consider increased work of breathing and its effect on nutritional support, growth and rehabilitation
Age	• Consider patient size and available non-invasive support options. • Smaller patients have lower reserve, making post-extubation challenges like secretion management and lung recruitment top priorities after extubation.

LVAD; Left Ventricular Assist Device, SpO2; Pulse Oxygen Saturation, FiO2; Fraction Inspired Oxygen, PEEP; Positive End Expiratory Pressure, ScvO2; Central Venous Oxygen Saturation, NIRS; Near-infrared spectroscopy.

### Strategies for optimizing LVAD support in patients with right ventricular failure

In the early postoperative period, fluid shifts and hemodynamic instability can make it challenging to differentiate between LVAD performance issues, RV failure, hypovolemia, or tamponade.^105^ If echocardiography confirms adequate LV decompression with a midline septum, management should prioritize RV support rather than assuming inadequate LVAD function. Caution is needed in smaller pediatric patients to prevent excessive left-sided decompression and septal leftward shift from the LVAD, which can worsen TR and trigger RVF. Significant bleeding or fluid loss may require temporary LVAD speed reduction as long adequate CO is maintained. If stability is not achieved, repeat echocardiography, advanced hemodynamic monitoring, or catheterization is recommended to identify the underlying cause.^105^

In cases of suspected RVF with low LVAD flows, echocardiographic assessment should evaluate left atrial and ventricular decompression, aortic valve opening, septal position, RV dilation, TR and cannula positioning (Table 8). Frequent evaluation of LV unloading is crucial, as optimizing LV end-diastolic pressure improves RV afterload. Persistent LV dilation, abnormal septal positioning, or excessive antegrade aortic valve flow suggests inadequate LV unloading, warranting gradual LVAD speed adjustments with monitoring of hemodynamic and echocardiographic parameters. Failure to achieve expected VAD flows or adequate cardiac index despite optimized therapies may indicate refractory RVF, requiring mechanical circulatory support.

**Table 8:** Echocardiographic Assessment for Clinical Concern of Right Ventricular Failure (RVF) Leading to Low Left Ventricular Assist Device (LVAD) Flows

Echocardiographic Assessment	Criteria/Considerations
Adequacy of LA and LV Decompression	goal is to ensure adequate LA and LV decompression to unload the pulmonary circulation and minimize PVR. • **Signs of inadequate LVAD decompression:** Persistent pulmonary edema or pleural effusions may indicate insufficient LV unloading. • **LVAD speed titration:** Caution is needed when increasing LVAD speeds, with close monitoring of the IVS position, RV dilation, and TR, as these factors can contribute to worsening RV failure. • **Challenges in LVAD optimization:** Difficulty in achieving the desired LVAD support level may be due to anatomical factors or suboptimal inlet positioning. Thorough evaluation with imaging and hemodynamic assessment is required to identify and address the underlying cause.
Midline/Neutral IVS	• The IVS should remain in a midline or neutral position to avoid RV anatomical changes which can lead to TR and inadequate ejection. • Any LVAD titrations should be complemented by echo assessment of IVS.
Degree of RV Dilation and TR	• RV performance should be evaluated through assessment of contractility, chamber dilation and the extent of TR. • TAPSE can be used as objective measurement of response to decongestive or inotropic therapies.

LVAD; Left Ventricular Assist Device, LA; Left Atrium, LV; Left Ventricle, RV; Right Ventricle, TR; Tricuspid Regurgitation, IVS; Interventricular Septum, RVF; Right Ventricular Failure, TAPSE; tricuspid annular plane systolic excursion

#### Mechanical circulatory support for RVF

For refractory RVF, mechanical support with a RVAD should be considered. Registry data indicates RVAD support is necessary in about 5% of pediatric LVAD patients.^1^ The choice of support depends on RVF acuity, resources, and institutional experience. In hyperacute postoperative instability, full ECMO is often the quickest stabilization method, bridging to RV recovery or durable surgical support. For acute RVF, paracorporeal temporary devices, such as RA to PA cannulation, can provide effective RV support.

For progressive RVF, an RVAD may be preferred over ECMO, with device selection based on patient size and anticipated support duration. Temporary support options include paracorporeal devices like PediMAG™, CentriMAG™ (Abbott, Pleasanton, CA), or RotaFlow (Getinge, Getinge, Sweden), surgically implanted with inflow cannulas in the RA and outflow cannulas in the PA. Larger patients may benefit from transcatheter devices like Impella RP (Abiomed, Danvers, MA) or Protek Duo (LivaNova, London, UK), deployed in the catheterization lab to avoid repeat sternotomy.

In patients unlikely to recover RV function, such as those with biventricular noncompaction, severe RV dilation, or restrictive cardiomyopathy, durable VAD support is recommended. The choice between atrial or ventricular cannulation depends on anatomical features, particularly to avoid compromising the tricuspid valve. Larger patients may be candidates for implantable RVADs, potentially allowing ICU discharge, while smaller patients may require long-term paracorporeal support, often necessitating extended ICU stays awaiting heart transplantation.

RVAD settings should be titrated to complement LVAD support, aiming to optimize systemic perfusion while avoiding pulmonary congestion. Because the RVAD supports a lower-resistance pulmonary circuit, flows typically target 60–80% of LVAD output. Effective cardiac output can be achieved even when RVAD and LVAD cardiac indices differ, reflecting their distinct mechanical demands. Key titration goals include maintaining a CVP of 8–12 mmHg to optimize preload without causing venous congestion, and monitoring for signs of pulmonary congestion and use of real-time echocardiography. RV size and septal position should be assessed regularly, along with systemic perfusion markers and lactate clearance. A multidisciplinary team, including surgical, critical care, and heart failure specialists, should integrate hemodynamic, imaging, and device data in real time to guide individualized, balanced support.

The decision to initiate “elective” RVAD support at the time of LVAD implantation remains a controversial aspect of VAD care. While some adult studies suggest improved outcomes with early RVAD insertion compared to delayed support,^106^, ^107^, ^108^ in pediatrics, practices vary widely, reflecting ongoing limitations in accurately predicting RV failure. Continued efforts to refine early diagnostics and foster inter-institutional collaboration are critical to optimizing timing and patient selection for RVAD support.

Finally, consideration and timing of RVAD explantation in the setting of ongoing LVAD support requires a stepwise, multidisciplinary approach. Weaning should be considered when the patient demonstrates hemodynamic stability without inotropic or pulmonary vasodilator support, preserved end-organ function, normalized fluid status with minimal diuretic dependence, and stable CVP (typically 8–12 mmHg). Echocardiography should show improved RV function, midline septal position, and no significant tricuspid regurgitation. RVAD flow is gradually reduced in small increments (e.g., 0.5 L/min), with close monitoring of systemic perfusion and RV function. If stability is maintained at low RVAD flow (e.g., ≤1 L/min) for several days, explantation may be considered. At this stage, a comprehensive hemodynamic assessment in the catheterization lab, including a trial off RVAD support, can provide valuable insight into the patient’s readiness for explantation. This process should involve coordinated decision-making among surgical, critical care, and heart failure teams to optimize safety and outcomes.

## Conclusion

RVF is a frequent and significant complication following LVAD placement, necessitating timely recognition and targeted management to prevent end-organ damage and improve outcomes. Successful treatment relies on optimizing preload, afterload, and contractility while monitoring hemodynamic parameters and organ function closely. When medical therapies fail, mechanical support with an RVAD becomes essential to stabilize the patient and support recovery. A consensus summary of key management principles is outlined in Table 9.

**Table 9:** Consensus Opinion on Peri-operative Care of the Right Ventricle After LVAD Placement. The table presents 21 peri-operative takeaway points derived from the manuscript and presented by the lead and senior authors to the co-author group predominantly composed of cardiac intensivists, alongside representation from cardiac surgery and transplant/heart failure. Consensus views among the authorship group are rated on a scale from 1 to 5 (1 = strongly disagree, 5 = strongly agree), indicating the percentage of agreement among authors and the average strength of agreement

Statement	Average Score	Strongly Disagree	Somewhat Disagree	Neither Agree Nor Disagree	Somewhat Agree	Strongly Agree
Statement 1: Obtain echocardiography to evaluate RV function, tricuspid and mitral regurgitation. When feasible, perform right heart catheterization to assess PVR and calculate PAPi.	4.5	0 (0%)	1 (6%)	0 (0%)	5 (31%)	10 (63%)
Statement 2: Optimize RV function before LVAD implantation by targeting a CVP of 8–12 mmHg through optimizing inotropic support, volume status, considering inhaled nitric oxide (iNO) for RV afterload reduction if evidence of elevated PVR, controlling arrhythmias, optimization of liver function.	4.1	0 (0%)	3 (19%)	0 (0%)	6 (37%)	7 (44%)
Statement 3: In the immediate post-LVAD period, cautiously optimize preload with a target CVP of 8–10 mmHg. Avoid large-volume resuscitation to prevent RV overdistention and dysfunction.	4.6	0 (0%)	0 (0%)	0 (0%)	6 (37%)	10 (63%)
Statement 4: In high-risk patients with evidence of elevated PVR, prophylactically initiate iNO and consider early initiation of pulmonary vasodilators	4.9	0 (0%)	0 (0%)	0 (0%)	1 (6%)	15 (94%)
Statement 5: Consider surgical repair of anatomical TV lesions during LVAD implantation	3.7	0 (0%)	2 (12%)	3 (19%)	9 (57%)	2 (12%)
Statement 6: Postoperatively, monitor TR severity in conjunction with CVP trends, RV dilation, and signs of venous congestion to guide need for TV intervention.	4.3	0 (0%)	0 (0%)	2 (12%)	8 (47%)	6 (37%)
Statement 7: Optimize and closely monitor RV preload to avoid systemic venous congestion.	4.8	0 (0%)	0 (0%)	0 (0%)	3 (18%)	13 (76%)
Statement 8: Closely monitor cardiac output and perfusion indices including urine output, central venous saturations, NIRS, lactate, liver function tests and renal markers and evaluate aggressively for RV failure if compromised.	5	0 (0%)	0 (0%)	0 (0%)	0 (0%)	16 (100%)
Statement 9: Actively reduce venous congestion to prevent or mitigate CRS.	4.7	0 (0%)	0 (0%)	0 (0%)	5 (31%)	11 (69%)
Statement 10: Monitor fluid balance closely and initiate diuresis early to avoid fluid overload.	4.7	0 (0%)	0 (0%)	0 (0%)	5 (31%)	11 (69%)
Statement 11: Place dialysis access preemptively in high-risk patients to ensure timely initiation of renal replacement therapy.	3.8	0 (0%)	2 (12%)	3 (18%)	6 (37%)	4 (25%)
Statement 12: Use inodilators to augment RV function post-LVAD.	4.5	0 (0%)	0 (0%)	2 (12%)	4 (25%)	10 (63%)
Statement 13: Avoid hypotension and maintain goal perfusion pressures to preserve coronary perfusion pressures for the RV.	4.8	0 (0%)	0 (0%)	0 (0%)	3 (18%)	13 (81%)
Statement 14: Aggressively treat any arrhythmias to optimize RV output and LVAD filling.	4.6	0 (0%)	0 (0%)	0 (0%)	6 (37%)	10 (63%)
Statement 15: Titrate ventilator settings to optimize functional residual capacity and minimize elevations in PVR.	4.9	0 (0%)	0 (0%)	0 (0%)	2 (12%)	14 (88%)
Statement 16: Avoid lung overdistension or de-recruitment and initiate early rehabilitation to facilitate successful extubation.	4.9	0 (0%)	0 (0%)	0 (0%)	1 (6%)	15 (94%)
Statement 17: Titrate LVAD support to achieve adequate CO and optimal RV afterload through direct LV unloading.	4.9	0 (0%)	0 (0%)	0 (0%)	1 (6%)	15 (94%)
Statement 18: Utilize echocardiography during LVAD titrations to optimize LVAD support and avoid LV dilation, abnormal septal positioning, excessive antegrade aortic valve flow or the lack of.	4.8	0 (0%)	0 (0%)	0 (0%)	3 (18%)	13 (76%)
Statement 19: Do not delay RVAD initiation in refractory RVF marked by persistently high CVP, systemic venous congestion, evidence of low CO and organ dysfunction.	4.9	0 (0%)	0 (0%)	0 (0%)	2 (12%)	14 (88%)
Statement 20: Use peripheral VA ECMO for hyperacute presentations of RVF.	4.5	0 (0%)	1 (6%)	0 (0%)	5 (31%)	10 (63%)
Statement 21: Consider use of paracorporeal RVADs for acute support, and durable devices in patients unlikely to recover RV function.	4.7	0 (0%)	0 (0%)	0 (0%)	5 (31%)	11 (69%)

## Funding sources

There was no support received for the writing of this review article. The review however was endorsed by the Advanced Cardiac Therapies Improving Outcomes Network (ACTION). ACTION in itself receives funding support from Berlin Heart EXCOR (Berlin, Germany), Abbott Laboratories (Chicago, IL), Medtronic (Minneapolis, MN), Abiomed (Danvers, MA), Syncardia (Tucson, AZ), Bayer (Leverkusen, Germany).

## Declaration of Competing Interest

The authors declare the following financial interests/personal relationships which may be considered as potential competing interests: There was no support received for the writing of this review article. The review however was endorsed by the Advanced Cardiac Therapies Improving Outcomes Network (ACTION). ACTION in itself receives funding support from Berlin Heart EXCOR (Berlin, Germany), Abbott Laboratories (Chicago, IL), Medtronic (Minneapolis, MN), Abiomed (Danvers, MA), Syncardia (Tucson, AZ), Bayer (Leverkusen, Germany). If there are other authors, they declare that they have no known competing financial interests or personal relationships that could have appeared to influence the work reported in this paper.
